# Prospective association between plasma amino acids and healthy aging in older adults

**DOI:** 10.1111/joim.20105

**Published:** 2025-06-12

**Authors:** Damián González‐Beltrán, Humberto Yévenes‐Briones, Alberto Lana, Juan Cárdenas‐Valladolid, Miguel Ángel Salinero‐Fort, Fernando Rodríguez‐Artalejo, Esther Lopez‐Garcia, Francisco Félix Caballero

**Affiliations:** ^1^ Department of Preventive Medicine and Public Health Universidad Autónoma de Madrid Madrid Spain; ^2^ CIBER of Epidemiology and Public Health Instituto de Salud Carlos III Madrid Spain; ^3^ Department of Medicine Universidad de Oviedo/ISPA Oviedo Spain; ^4^ Dirección General de Investigación y Docencia Consejería de Sanidad de la Comunidad Autónoma de Madrid Madrid Spain; ^5^ Enfermería Universidad Alfonso X El Sabio Villanueva de la Cañada Spain; ^6^ Subdirección General de Investigación Sanitaria Consejería de Sanidad Fundación de Investigación e Innovación Sanitaria de Atención Primaria Madrid Spain; ^7^ Red de Investigación en Servicios de Salud en Enfermedades Crónicas Grupo de Envejecimiento y Fragilidad de las personas mayores IdIPAZ Madrid Spain; ^8^ IMDEA‐Food Institute CEI UAM+CSIC Madrid Spain

**Keywords:** amino acids, healthy aging, lifestyle behaviors, metabolomic profiling, older adults

## Abstract

**Background:**

Most studies have compared plasma amino acids profiling across different age groups using a cross‐sectional design, but no previous research has assessed the relationship between specific amino acid species and healthy aging.

**Objectives:**

This study aims to explore the relationship between plasma concentrations of nine amino acids and healthy aging in an older Spanish population.

**Methods:**

This longitudinal study uses data from the Seniors‐ENRICA 2 Spanish cohort, which comprises community‐dwelling individuals aged 65 and older. Plasma amino acid concentrations were measured at baseline and after a 5‐year follow‐up period (*n* = 859). Healthy aging has been defined as the delay on the onset of chronic conditions, optimal physical functioning, and no cognitive impairment. Multilevel mixed effect logistic models were used to examine the prospective association proposed, after adjusting for age, sex, socioeconomic status, and lifestyle behaviors.

**Results:**

The baseline mean age of the participants was 70.9 years (standard deviation [SD] = 4.0), and 51.6% were men. In the fully adjusted models, lower plasma concentrations of alanine [odds ratios per 1‐SD increase (95% confidence interval) = 0.78 (0.72, 0.86)], isoleucine [0.70 (0.63, 0.78)], leucine [0.78 (0.71, 0.86)], and valine [0.79 (0.71, 0.86)] were prospectively associated with healthy aging (*p*‐value < 0.001 in all cases). No significant associations were observed for glutamine, glycine, histidine, and aromatic amino acids.

**Conclusion:**

Lower concentrations of alanine and branched‐chain amino acids were prospectively associated with healthy aging in the older population.

## Graphical abstract

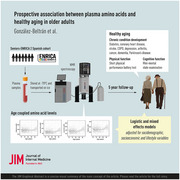

**Abbreviations**
AAA: aromatic amino acid
AHEI: alternative healthy eating index
BCAA: branched‐chain amino acid
BMI: body mass index
CI: confidence interval
CV: coefficient of variation
ICPC‐2: international classification of primary health‐2nd edition
MELM: mixed‐effects logistic model.
MET: metabolic equivalent task
MMSE: mini‐mental state examination
NMR: nuclear magnetic resonance
OR: odds ratio
SD: standard deviation
SPPB: short physical performance battery

## Introduction

Achieving good health and well‐being as we age requires individual and efforts throughout life, along with an environment that supports a healthy lifestyle. According to the World Health Organization, healthy aging is the process of developing and maintaining functional ability to enable well‐being in older age [1]. Healthy aging has been recently operationalized by three criteria: (i) optimal physical and cognitive functioning, (ii) delay of the onset of chronic diseases, and (iii) maximally extended lifespan [2].

The hallmarks of aging are biological mechanisms that explain the acceleration of the process of aging [3]. Metabolomic studies on aging have sought potential biomarkers of age‐related diseases [4, 5]. Metabolites, which are low‐molecular‐weight intermediates in biological systems, can be influenced by multiple factors, including psychological function, endocrine function, inflammation, physical capability, and cognitive function [6]. Several studies have already found associations between a wide range of metabolites, including carbohydrates, carnitines, biogenic amines, and lipids, and the process of aging [7, 8].

Amino acids profiling has also been studied. Specifically, current evidence suggests that tyrosine levels tend to rise with age [9, 10, 11]. Conversely, plasma tryptophan typically decreases with age [9, 10, 12]. Additionally, some studies indicated that older adults generally display high concentrations of leucine, isoleucine, and valine, also known as branched‐chain amino acids (BCAAs) [13, 14, 15]. Most studies have compared plasma amino acids profiling across different age groups using a cross‐sectional design [13, 15–19], but previous research has not assessed to date the relationship between specific amino acid species and healthy aging. In order to identify potential biomarkers of healthy aging, the aim of this study was to explore the longitudinal association between plasma amino acid profiling and healthy aging within a Spanish population aged 65 years and during a 5‐year follow‐up period.

## Methods

### Design and population

The analytical sample of this longitudinal study was selected from the Seniors‐ENRICA 2, a Spanish cohort of older adults, which was established between 2015 and 2017 (baseline period) using sex‐ and district‐stratified random sampling methods [20, 21]. The cohort comprised non‐institutionalized, community‐dwelling individuals over 65 years living in Madrid and four nearby towns and holding a national healthcare card. In 2019 and 2022, two follow‐up phases were conducted. Computer‐assisted telephone interviews were performed to collect sociodemographic and socioeconomic characteristics, lifestyle behaviors, and health‐related conditions, and two home visits were subsequently conducted to perform a physical examination and obtain biological samples. Plasma samples were collected by trained nurses at the participant's home under standardized conditions. A total of 3273 participants were initially interviewed in the baseline phase of the Seniors‐ENRICA 2 cohort. Of them, 1227 completed the whole follow‐up period. As the characteristics of this study, we were interested on the participants with plasma samples available during the follow‐up (*n* = 876, a 71.4% of those who completed the whole follow‐up period). Finally, after considering those participants with information in all study variables, our analytical sample comprised 859 participants, a 98.1% of those with plasma samples.

When dividing the number of subjects interviewed at baseline (*n* = 3273) by the total number of people initially invited to participate in the Seniors‐ENRICA 2 cohort, the response rate obtained was 38%. This cohort used similar instruments and procedures to the described for the Seniors‐ENRICA 1 [22], which reported a response rate of 51%. Written informed consent was obtained from all participants, and the study protocol was approved by the Clinical Research Committee of “La Paz” University Hospital (PI‐1793, PI‐3554) in Madrid, Spain.

### Healthy aging

Healthy aging definition has been based on the concept of healthy biomedical aging [23]. Specifically, healthy aging was defined as a dichotomous variable and categorized as healthy/unhealthy aging. Healthy aging was considered being free of nine chronic conditions, optimal physical functioning, and no cognitive impairment.

Information on diagnosed chronic diseases was obtained through the primary care electronic medical records (using codes from the International Classification of Primary Health, 2nd edition (ICPC‐2). The nine chronic diseases considered were diabetes (T89, T90), coronary heart disease (K74‐K76), stroke (K89‐K91), chronic obstructive pulmonary disease (R95), depression (P76), arthritis (L88), cancer (A79, D74‐D76, D77, L71, R84, R85, S77, T71, U75‐U77, X75‐X77, Y77), dementia (P70), and Parkinson's disease (N87). These conditions are among the leading causes of global burden diseases in high‐income countries [24]. The Research Central Commission of Madrid Regional Health Service provided permission to access participants’ electronic clinical records. The quality of the primary care electronic clinical records has previously been validated for research purposes [25].

Physical functioning was measured by physical examination, using the short physical performance battery test [26, 27]. Optimal physical functioning was defined as a score higher than nine in the SPBB test. Cognitive impairment was self‐reported by completing the mini‐mental state examination (MMSE) test; MMSE scores ≥24, which is the cut‐off recommended for people over 65 years [28], indicated cognitive impairment.

### Amino acid profiling

Plasma samples were stored at −70°C at the Universidad Autónoma de Madrid Biobank until processing and shipped to the laboratory on ice for analysis at the Nightingale Health laboratory (Nightingale Health Ltd). Plasma concentrations of amino acids were quantified using nuclear magnetic resonance spectroscopy based on metabolomics [29], with results expressed in millimoles per liter (mmol/L) and transformed into micromolar (µM) for the aims of this study. Nine amino acid species were measured: alanine, glutamine, glycine, histidine, isoleucine, leucine, valine, phenylalanine, and tyrosine. The reproducibility of assay among stored plasma samples showed consistent values, in terms of the interbatch coefficient of variation, with values ranging from 2.5% (alanine) to 9.9% (histidine). The amino acids considered included BCAAs (isoleucine, leucine, and valine) and aromatic amino acids (AAAs) (phenylalanine and tyrosine).

### Lifestyle behaviors

Adherence to a healthy lifestyle was evaluated based on five patterns: (i) diet quality, assessed through the alternative healthy eating index‐2010 [30], which is based on the intake of 11 food groups and nutrients (vegetables, fruits, whole grains, sugar‐sweetened beverages or fruit juice, red/processed meat, sodium, nuts and legumes, polyunsaturated fatty acids, long‐chain fats, trans fat, and alcohol), with a total score ranging from 0 to 110; higher scores indicate a healthier diet. Diet quality was classified in tertiles, representing low, medium, and high diet quality, respectively; (ii) physical activity was assessed using the validated EPIC physical activity questionnaire [31], quantified in metabolic equivalent tasks (METs), and stratified into tertiles based on weekly hours spent (METs h/week) on each of the following activities: walking, cycling, gardening, do‐it‐yourself activities at home, playing sports and climbing stairs; (iii) smoking status was categorized as never‐ and current or former‐ smokers; (iv) body mass index (BMI) was calculated as weight (kg) divided by squared height (m^2^), measured under standardized conditions by physical examination, and categorized into tertiles for analytical purposes; and (v) optimal sleep duration was obtained by participant response to the question “How many hours do you usually sleep at night and during the day?.” A healthy sleep pattern was defined as sleeping 7–8 h/day [32].

### Sociodemographic and socioeconomic characteristics

Participants were asked for sex and age. Educational attainment (university studies, secondary school, and primary education or less) and householder's occupational category were considered socioeconomic indicators. The categorization proposed by Lostao et al. [33] for the Spanish population was used for defining householder's occupational category, considering the latest occupation for those retired participants: (i) professionals and managers (professionals and employers‐managers); (ii) lower non‐manual workers (clerks and administrative personnel, service and sales workers, self‐employed workers, and supervisors); (iii) skilled manual workers; and (iv) unskilled manual workers (semiskilled manual workers and unskilled manual workers).

### Statistical analysis

A descriptive analysis of the characteristics of the population based on healthy and unhealthy aging was conducted, using categorical variables through the distribution of absolute and relative frequencies. Quantitative variables were represented by their mean and standard deviation (SD). Geometric means and 95% confidence intervals (CIs) for the nine amino acid concentrations were computed at baseline and after the two follow‐up periods.

Logistic regression models were used to assess the 5‐year association between the amino acids profiling and unhealthy aging (defined as presence of chronic conditions, non‐optimal physical functioning, or cognitive impairment), with odds ratios (ORs) being estimated per 1‐SD increase. Model 1 was adjusted for age, sex, educational attainment, and householder's occupational category, whereas Model 2 was additionally adjusted for lifestyle covariates, including diet quality, physical activity, smoking status, BMI, and optimal sleep duration. Participants who already presented unhealthy aging at baseline were excluded from these logistic regression models.

Furthermore, multilevel mixed‐effects logistic models (MELMs) were employed to use the information provided by repeated measures and to examine then the prospective association between amino acid concentrations and healthy aging. Random intercepts were considered in the multilevel MELM (see details in the Appendix section) to take into account that the baseline value is different and the repeated observations are correlated for each individual [34]. Data collected at baseline and at the two follow‐up phases were considered in the multilevel MELM, which is a method for binary outcomes, enabling the estimation of population‐average effect of covariates measured at both participants and cluster level [35]. Fixed‐effect coefficients can be interpreted as in a standard logistic regression model and were reported as ORs, together with 95% CI. Separated multilevel MELM were fitted for each of the nine amino acids. The models were adjusted for age, sex, socioeconomic variables (educational attainment and householder's occupational category), BMI, smoking status, physical activity, and optimal sleep duration.

To reduce potential multicollinearity and to assess the independent relationship of each amino acid with healthy aging, models were conducted separately for each amino acid. Mean correlation among the nine plasma concentrations of amino acids considered was 0.31. Specifically, mean correlation among the three BCAAs was 0.88, whereas the mean correlation among the two AAAs was 0.50.

As diet quality was not measured in the follow‐up phases, it was not included as a covariate in the multilevel MELM approach. Therefore, diet quality‐stratified multilevel MELM were conducted to examine the potentially different association between amino acids and healthy aging by baseline diet quality (low, moderate, and high). A local polynomial smooth plot was used to describe trends through the relationship between plasma amino acids and age, especially in contexts where the amino acids concentrations do not follow a linear trend, needing flexibility to capture local variations in age. Statistical analyses were conducted using Stata version 18 (StataCorp) and R (version 4.4.0) for graph analysis.

## Results

The baseline characteristics of the analytical sample are shown in Table 1. The mean age of participants included in the analytical sample (*n* = 859) was 70.9 (SD = 4.0) years, and 51.8% of the participants were men. Healthy older adults (47.0% of the sample) had a mean age of 70.5 years (SD = 3.9), whereas those unhealthy had a mean age of 71.3 years (SD = 4.0). Significant differences between both subgroups were not observed in terms of sex, educational attainment, and householder's occupational category. Regarding BMI at baseline, significantly higher values were observed for the unhealthy participants (27.9 ± 4.5 vs. 26.7 ± 3.8; *p*‐value < 0.001).

**Table 1:** Baseline characteristics of the analytical sample based on healthy and unhealthy aging (n = 859).

Variables^a^	Healthy aging^d^ (*n* = 404)	Unhealthy aging^e^ (*n* = 455)	*p*‐value^b^
**Sociodemographic characteristics**			
Age (years)	70.5 ± 3.9	71.3 ± 4.0	0.002
Men	211 (52.3)	232 (51.0)	0.717
**Socioeconomic status**			
Educational attainment			0.412
University	110 (27.3)	142 (31.2)	
Secondary school	195 (48.3)	212 (46.6)	
Primary education or less	99 (24.5)	101 (22.2)	
Householder's occupational category			0.057
Professionals and managers	122 (30.2)	128 (28.1)	
Lower non‐manual workers	147 (36.4)	169 (37.1)	
Skilled manual workers	104 (25.7)	99 (21.8)	
Unskilled manual workers	31 (7.7)	59 (13.0)	
**Lifestyle covariates**			
Physical activity (METs h/week)	69.9 ± 34.6	68.2 ± 38.3	0.503
BMI (kg/m^2^)	26.7 ± 3.8	27.9 ± 4.5	<0.001
Never smokers	215 (53.2)	219 (48.1)	0.137
Optimal sleep duration	168 (41.6)	176 (36.7)	0.386
AHEI‐2010 score	63.8 ± 9.5	63.6 ± 9.8	0.778
**Plasma amino acids** (µM)^c^, (95% CI)			
Alanine	368.8 (360.9, 376.8)	383.7 (375.9, 391.7)	0.009
Glutamine	639.5 (633.2, 645.8)	645.9 (639.1, 652.7)	0.180
Glycine	189.6 (184.3, 195.0)	182.9 (177.8, 188.1)	0.077
Histidine	69.9 (69.1, 70.8)	69.4 (68.6, 70.2)	0.361
Isoleucine	53.9 (52.6, 55.2)	59.4 (57.9, 61.0)	<0.001
Leucine	125.3 (122.9, 127.8)	130.6 (128.1, 133.2)	0.004
Valine	241.8 (238.2, 245.5)	252.0 (248.2, 255.8)	<0.001
Phenylalanine	56.7 (55.8, 57.7)	56.9 (56.0, 57.9)	0.757
Tyrosine	69.9 (68.8, 71.0)	69.7 (68.7, 70.8)	0.841

Abbreviations: AHEI: Alternative Healthy Eating Index; BMI: body mass index; CI: confidence interval; MET: metabolic equivalent task; MMSE: Mini‐Mental State Examination; SD: standard deviation; SPPB: Short Physical Performance Battery.

^a^ Mean (SD) for continue variables and *n* (%) for categorical ones.

^b^ *p*‐values were obtained after applying an unpaired *t* test or chi‐square test, depending on the type of variable.

^c^ Geometric means (95% confidence interval) of plasma amino acids (*p*‐values were obtained after applying an unpaired *t* test over the log‐transformed amino acids).

^d^ No presence of chronic conditions, optimal physical functioning (SPPB score >9), or no cognitive impairment (MMSE score ≥24).

^e^ Presence of chronic conditions, non‐optimal physical functioning (SPPB score ≤9), or cognitive impairment (MMSE score <24).

Table S1 showed baseline sociodemographic, socioeconomic, and lifestyle characteristics for the analytical sample (*n* = 859) and those participants in the 5‐year follow‐up period who did not have plasma samples measured or observed values in the remaining study variables (*n* = 368). A lower mean age (70.9 ± 4.0 vs. 71.6 ± 4.3; *p*‐value = 0.008) and BMI (27.3 ± 4.2 vs. 28.1 ± 4.7; *p*‐value = 0.004) were observed in the analytical sample. When comparing the baseline characteristics of the analytical sample with the information collected from those who were interviewed in the baseline phase of the Seniors‐ENRICA‐2 cohort but did not participate in the follow‐up phases (*n* = 2414), a significantly higher proportion of men, people with university studies, and professionals and managers was observed in the analytical sample (Table S2), together with significantly higher mean values in physical activity and adherence to diet quality.

The geometric means of plasma amino acids at baseline and after the two follow‐up periods are shown in Table 2. Plasma concentrations of glutamine increased consistently across the follow‐up period, with geometric means (95% CI) of 642.9 µM (638.2, 647.5) at baseline, 660.9 µM (656.2, 665.7) in 2019, and 676.1 µM (671.2, 681.0) in 2022. In contrast, a general increase trend was not observed for plasma concentrations of alanine, glycine, histidine, and BCAAs and AAAs.

**Table 2:** Geometric means (95% confidence interval) of plasma concentrations of amino acids (µM) at baseline and after the two follow‐up periods in older adults (n = 859).

	Baseline		2019		2022	
Amino acid species (µM)						
Alanine	376.6	(371.0, 382.3)	359.1	(354.3, 364.0)	361.5	(356.9, 366.2)
Glutamine	642.9	(638.2, 647.5)	660.9	(656.2, 665.7)	676.1	(671.2, 681.0)
Glycine	186.0	(182.4, 189.8)	179.5	(175.7, 183.4)	187.1	(183.3, 190.9)
Histidine	69.6	(69.0, 70.2)	67.0	(66.3, 67.8)	72.8	(72.3, 73.4)
BCAAs						
Isoleucine	56.8	(55.7, 57.8)	55.5	(54.5, 56.5)	63.1	(62.1, 64.1)
Leucine	128.1	(126.3, 129.9)	122.1	(120.4, 123.8)	132.9	(131.2, 134.6)
Valine	247.1	(244.5, 249.8)	242.2	(239.7, 244.9)	247.6	(245.0, 250.2)
AAAs						
Phenylalanine	56.8	(56.2, 57.5)	54.3	(53.7, 54.8)	56.8	(56.2, 57.3)
Tyrosine	69.8	(69.0, 70.6)	67.7	(67.0, 68.5)	72.2	(71.5, 73.0)

Abbreviations: AAAs, aromatic amino acids; BCAAs, branched‐chain amino acids.

Logistic regression models for assessing the relationship between baseline amino acids profiling and unhealthy aging over the 5‐year follow‐up are shown in Table 3. After excluding participants with unhealthy aging at baseline and after adjusting for age, sex, socioeconomic status (educational attainment and householder's occupational category), and lifestyle behaviors, significant associations with unhealthy aging were found for higher plasma concentrations of alanine [OR per 1‐SD increase (95% CI) = 1.43 (1.12, 1.82), *p*‐value = 0.004], isoleucine [OR = 1.35 (1.00, 1.82), *p*‐value = 0.049], and tyrosine [OR = 1.31 (1.04, 1.66), *p*‐value = 0.022].

**Table 3:** Logistic regression models for assessing the 5‐year relationship between baseline amino acids profiling and unhealthy aging, after excluding participants with unhealthy aging at baseline (n = 404).

	Model 1			Model 2		
	OR^a^	(95% CI)	*p*‐value	OR^a^	(95% CI)	*p*‐value
Amino acid species (µM)						
Alanine	1.47	(1.16, 1.86)	0.001	1.43	(1.12, 1.82)	0.004
Glutamine	1.13	(0.89, 1.43)	0.304	1.17	(0.92, 1.48)	0.208
Glycine	1.11	(0.87, 1.41)	0.408	1.16	(0.90, 1.48)	0.244
Histidine	0.86	(0.68, 1.08)	0.201	0.86	(0.68, 1.09)	0.206
BCAAs						
Isoleucine	1.43	(1.07, 1.91)	0.016	1.35	(1.00, 1.82)	0.049
Leucine	1.28	(0.98, 1.67)	0.067	1.21	(0.92, 1.59)	0.179
Valine	1.30	(1.01, 1.68)	0.039	1.22	(0.93, 1.59)	0.145
AAAs						
Phenylalanine	1.28	(1.00, 1.64)	0.048	1.24	(0.97, 1.60)	0.091
Tyrosine	1.36	(1.09, 1.71)	0.008	1.31	(1.04, 1.66)	0.022

*Note*: Model 1 adjusted for baseline age, sex, educational attainment, and householder's occupational category. Model 2 was further adjusted for baseline physical activity (tertiles of METs‐h/week), BMI (tertiles of kg/m^2^), smoking status, optimal sleep duration, and AHEI‐2010 score (tertiles).

Abbreviations: AAAs, aromatic amino acids; AHEI, Alternative Healthy Eating Index; BCAAs, branched‐chain amino acids; BMI, body mass index; CI, confidence interval; OR, odd ratio; SD, standard deviation.

^a^ Odds ratios were estimated by 1‐SD increase.

Fully adjusted multilevel MELM are shown in Table 4, and the results obtained, after considering repeated measures available in the follow‐up phases, showed that lower plasma concentrations of alanine and BCAAs were related to healthy aging, with *p*‐value < 0.001 in all cases. ORs per 1‐SD increase (95% CI) associated with healthy aging were 0.78 (0.72, 0.86) for alanine, 0.70 (0.63, 0.78) for isoleucine, 0.78 (0.71, 0.86) for leucine, and 0.79 (0.71, 0.86) for valine. No associations were observed for glycine, histidine, and AAAs.

**Table 4:** Multilevel mixed‐effect logistic models for assessing the prospective association between plasma amino acids (µM) and healthy aging, during the 5‐year follow‐up period (n = 859).

	Model 1			Model 2		
	OR^a^	(95% CI)	*p*‐value	OR^a^	(95% CI)	*p*‐value
Amino acid species (µM)						
Alanine	0.78	(0.72, 0.84)	<0.001	0.78	(0.72, 0.86)	<0.001
Glutamine	0.97	(0.90, 1.06)	0.542	0.91	(0.83, 0.99)	0.028
Glycine	1.02	(0.94, 1.11)	0.679	0.99	(0.91, 1.09)	0.897
Histidine	1.01	(0.93, 1.09)	0.839	1.01	(0.93, 1.10)	0.817
BCAAs						
Isoleucine	0.69	(0.63, 0.76)	<0.001	0.70	(0.63, 0.78)	<0.001
Leucine	0.76	(0.69, 0.83)	<0.001	0.78	(0.71, 0.86)	<0.001
Valine	0.75	(0.69, 0.82)	<0.001	0.79	(0.71, 0.86)	<0.001
AAAs						
Phenylalanine	0.90	(0.83, 0.98)	0.011	0.92	(0.84, 1.00)	0.055
Tyrosine	0.98	(0.91, 1.07)	0.700	1.02	(0.94, 1.12)	0.573

*Note*: Model 1 adjusted for age, sex, educational attainment, and householder's occupational category. Model 2 was further adjusted for physical activity (tertiles of METs‐h/week), BMI (tertiles of kg/m^2^), smoking status, and optimal sleep duration.

Abbreviations: AAAs, aromatic amino acids; BCAAs, branched‐chain amino acids; BMI, body mass index; CI, confidence interval; OR, odd ratio; SD, standard deviation.

^a^ Odds ratios were estimated by 1‐SD increase.

A stratified analysis by baseline diet quality is shown in Table 5. In general terms, the significant associations between lower plasma concentrations of alanine and BCAAs and healthy aging were observed in each of the strata considered. Regarding the remaining amino acid species, significant association between higher plasma concentrations of glycine [OR = 1.31 (1.11, 1.55), *p*‐value = 0.001] and histidine [OR = 1.23 (1.05, 1.43), *p*‐value = 0.010] and healthy aging were found for those with low diet quality. On the other hand, lower plasma concentrations of glutamine [OR = 0.82 (0.71, 0.96), *p*‐value = 0.015], histidine [OR = 0.85 (0.73, 0.99), *p*‐value = 0.038], and phenylalanine [OR = 0.83 (0.70, 0.97), *p*‐value = 0.020] were prospectively associated with healthy aging in those with high diet quality.

**Table 5:** Multilevel mixed‐effect logistic models for assessing the prospective association between plasma amino acids (µM) and healthy aging, by baseline diet quality^a^, during the 5‐year follow‐up period (n = 859).

	Low diet quality (*n* = 287)			Medium diet quality (*n* = 286)			High diet quality (*n* = 286)		
	OR^b^	(95% CI)	*p*‐value	OR^b^	(95% CI)	*p*‐value	OR^b^	(95% CI)	*p*‐value
Amino acid species (µM)									
Alanine	0.71	(0.60, 0.85)	<0.001	0.82	(0.71, 0.96)	0.011	0.79	(0.68, 0.93)	0.004
Glutamine	0.99	(0.84, 1.16)	0.878	0.92	(0.79, 1.07)	0.276	0.82	(0.71, 0.96)	0.015
Glycine	1.31	(1.11, 1.55)	0.001	0.87	(0.75, 1.02)	0.090	0.90	(0.76, 1.07)	0.225
Histidine	1.23	(1.05, 1.43)	0.010	1.02	(0.88, 1.18)	0.816	0.85	(0.73, 0.99)	0.038
BCAAs									
Isoleucine	0.72	(0.59, 0.87)	0.001	0.71	(0.59, 0.85)	<0.001	0.70	(0.59, 0.84)	<0.001
Leucine	0.80	(0.67, 0.96)	0.018	0.80	(0.67, 0.95)	0.013	0.76	(0.64, 0.90)	0.001
Valine	0.83	(0.70, 0.99)	0.037	0.75	(0.64, 0.89)	0.001	0.81	(0.69, 0.95)	0.011
AAAs									
Phenylalanine	0.93	(0.79, 1.10)	0.417	1.00	(0.87, 1.16)	0.995	0.83	(0.70, 0.97)	0.020
Tyrosine	1.02	(0.87, 1.19)	0.851	1.04	(0.90, 1.20)	0.611	1.03	(0.89, 1.20)	0.655

*Note*: Models were adjusted for age, sex, educational attainment, householder's occupational category, physical activity (tertiles of METs‐h/week), BMI (tertiles of kg/m2), smoking status, and optimal sleep duration.

Abbreviations: AAAs, aromatic amino acids; AHEI, Alternative Healthy Eating Index; BCAAs, branched‐chain amino acids; BMI, body mass index; CI, confidence interval; OR, odd ratio; SD, standard deviation.

^a^ Diet quality was defined as a three‐category variable based on tertiles of the AHEI‐2010 scores distribution.

^b^ Odds ratios were estimated by 1‐SD increase.

**Fig. 1:**
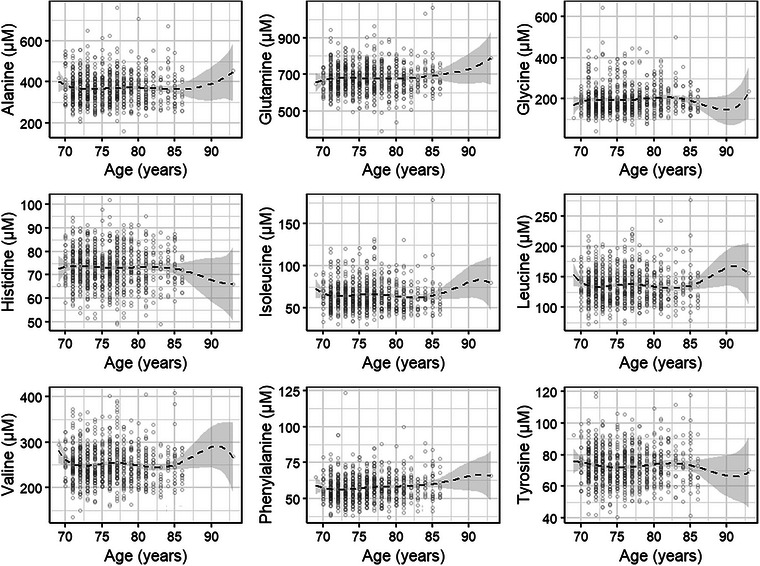
Local polynomial smooth plots for plasma amino acids (µM) and age, among those who participated in the follow‐up (n = 859); 95% confidence intervals.

Finally, local polynomial smooth plots are shown in Fig. 1. All species of BCAAs showed a negative association with age, meaning that their plasma concentrations tended to decrease as people aged. AAAs (phenylalanine and tyrosine) showed stable or slightly fluctuating concentrations with age, without significant associations. Lastly, glutamine showed a positive relationship with age, with concentrations increasing as people age, especially after 85 years.

## Discussion

In this cohort of older adults from Spain, we found that lower levels of alanine and BCAAs, such as isoleucine, leucine, and valine, were prospectively associated with healthy aging. These results would be in line with a previous article, which considered other health‐related outcome as multimorbidity [36], being the present study the first prospective one to show the potential role of plasma amino acids on a comprehensive definition of healthy aging in older population.

Alanine is an amino acid that plays a key role in the aging process [13, 14, 17, 18, 37–40]. Consistent with our results, a cohort study involving healthy older men reported lower levels of serum alanine [40]. Alanine is absorbed by the liver, where much of this non‐essential amino acid is converted to pyruvate, a key component of gluconeogenesis [41]. Similarly, metabolomic disorders such as Type 2 diabetes are associated with higher alanine concentrations compared to non‐diabetics individuals [38], which may be beneficial due to its potential to enhance both oxidative and non‐oxidative glucose metabolism, as well as stimulate insulin secretion [41].

Our results also showed that lower concentrations of BCAAs are related to healthy aging. Previous studies have observed higher plasma BCAAs in older men [14, 19, 42]. In fact, two reviews have shown that isoleucine and leucine levels decrease in the blood of aging individuals, making these reductions one of the most consistent aging markers across human studies [41, 43]. Several authors have also highlighted the association of BCAAs with a variety of lifestyle factors [15, 44]. Although no correlation was found between age and BCCA concentrations in older adults before and after resistance exercise training [45], other studies have showed that aging is associated with a reduced dietary intake of protein, leading to lower plasma BCAA levels during the aging process [43].

Previous research reported conflicting findings regarding plasma leucine levels in older adults. One study found that leucine levels were higher in older individuals than in younger ones [46], whereas others have reported lower levels in the aging population [17, 47]. Leucine is the primary BCAA regulating protein synthesis in skeletal muscle by the ribosome [41]. It has to increase the protein fractional synthetic rate in older adults, although it does not appear to significantly affect lean body mass or leg lean mass [43].

Glutamine is one of the most abundant amino acids in the human body, and it increases with adherence to combined healthy lifestyle behaviors [46]. These behaviors include maintaining a normal BMI [44, 48]. We observed a significant increase in plasma concentrations of glutamine after follow‐up, and a significant association between lower plasma concentrations of glutamine and healthy aging was only observed in those with adherence to a high diet quality. Previous studies reported that higher plasma glutamine concentrations were associated with higher levels of multimorbidity and risk of impaired lower‐extremity function in older adults [36, 49]. On the other hand, centenarian individuals have reported more abundant serum glutamine levels [50].

Regarding plasma glycine concentrations, our study revealed no significant association with healthy aging in the overall population, although a significant association between higher plasma concentrations of glycine and healthy aging was found in those adhered to a low diet quality. Glycine serves as precursor for several key metabolites, including creatine, glutathione, heme, purines, and porphyrins [51]. Although dietary supplementation with appropriate doses of glycine has proven effective in managing certain diseases and metabolic disorders [41], recent research has not found a relationship between plasma glycine levels and a healthy diet [44]. Moreover, several studies have reported higher concentrations in older women [14, 16, 19, 42], being one of the non‐essential amino acid that increases in abundance with aging [41].

In our study, we have not found a significant relationship between histidine and healthy aging in the overall population. However, when stratifying by baseline diet quality, we observed that higher plasma concentrations of histidine were prospectively related to healthy aging in those with low diet quality, whereas lower plasma concentrations were related to healthy aging in those adhered to a high diet quality. These results suggest that diet quality could moderate the relationship between plasma concentrations of histidine and healthy aging. A previous study observed a significant decrease in 3‐methylhistidine, a histidine analog, in participants who followed a Mediterranean diet [52].

Lastly, we did not find a significant association between the two AAAs analyzed (phenylalanine and tyrosine) and healthy aging when considering all the information available during the follow‐up period. However, a significant relationship between higher plasma concentrations of tyrosine and unhealthy aging was observed when excluding those participants with unhealthy aging at baseline. A previous study found that tyrosine levels tended to be lower in individuals with normal BMI [44], whereas plasma tyrosine levels can increase with age [13], a pattern consistent with our findings. We also observed a significant association between lower plasma concentrations of phenylalanine and healthy aging in those adhered to a high diet quality.

The strengths of this study included using a population‐based cohort where sociodemographic, socioeconomic, and lifestyle behaviors have been well characterized. Healthy biomedical aging has been conceptualized across three main domains (delay on the onset of chronic conditions, optimal physical functioning, and no cognitive impairment). The ICPC‐2 code was used to classify chronic conditions, whereas standardized and reliable instruments were used to characterize physical and cognitive decline. In addition, we employed multilevel MELM to assess the relationships between amino acid concentrations and healthy aging, incorporating longitudinal data from three different phases (baseline and follow‐up periods). The adjusted multilevel MELM also accounted for a wide range of sociodemographic, socioeconomic, and lifestyle variables as potential confounders. To avoid potential reverse causation, logistic regression models were conducted for assessing the 5‐year relationship between baseline amino acids profiling and unhealthy aging, after excluding those participants who already presented unhealthy aging at baseline.

Our findings should also be interpreted in the context of some limitations. First, we considered a set of nine amino acids separately, including BCAAs and AAAs, but other amino acids like tryptophan, which typically decreases with age [9, 10, 12], were not assessed. Diet quality was not measured during the follow‐up period of the study and was not included as an adjustment variable in the multilevel MELM; therefore, a stratified analysis based on the adherence to a diet quality was conducted to assess potential differences in the relationship between amino acids profiling and healthy aging. Potential measurement bias could be found as questionnaires were self‐administered. For example, diet was self‐reported, so certain misclassification and social desirability bias may have occurred. Finally, the study sample was recruited within the metropolitan area of Madrid, but, although it took into account the heterogeneity by sex and district, the representativeness of the Spanish older population cannot be assumed; moreover, due to the observational nature of the study design, causal inferences cannot be scientifically drawn from our results to wider populations [53].

In conclusion, our results suggest that lower concentrations of alanine and BCAAs were prospectively associated with healthy aging. The role of these amino acids as potential biomarkers for healthy aging in older adults should be confirmed in future studies. Increased research in metabolomics will further clarify the role of amino acids, enabling more effective health promotion, resource allocation, and preventive interventions to improve global health and longevity of older adults. Further research is needed to assess the trajectories of these amino acids on different cohorts of older adults and to explore how lifestyle behaviors influence the aging process.

## Author contributions

Damián González‐Beltrán performed statistical analyses and drafted the manuscript. Francisco Félix Caballero provided statistical expertise and supervised the work. Francisco Félix Caballero and Esther Lopez‐Garcia created the study concept and design. All authors reviewed the manuscript for important intellectual content, read, and approved the final manuscript.

## Conflicts of interest statement

The authors declare no conflicts of interest.

## Funding information

This study has been funded by four grants (PI 19/319, PI 19/665, PI 20/1040, PI 23/272) from the Instituto de Salud Carlos III (Spanish Ministry of Science and Innovation, Madrid, Spain, and European Regional Development Fund‐ERDF). DG‐B has received a predoctoral contract for the training of research personnel from Universidad Autónoma de Madrid (FPI‐UAM). The funding agencies had no role in study design, data analysis, interpretation of results, manuscript preparation, or in the decision to submit this manuscript for publication.

## Supporting information




**Table S1**. Baseline sociodemographic, socioeconomic, and lifestyle characteristics for the analytical and excluded samples.
**Table S2**. Baseline sociodemographic, socioeconomic, and lifestyle characteristics for the analytical sample and those participants initially interviewed in the baseline phase of the Seniors‐ENRICA‐2 cohort who did not participate in the follow‐up phases.

## Data Availability

Research data are not shared.
