# Supplementary material for: Prospective association between plasma amino acids and healthy aging in older adults
Source: J Intern Med. 2025 Jun 12;298(2):123–34. doi: 10.1111/joim.20105 (PMC12239062; doi:10.1111/joim.20105)
Supplement: Supplementary file 1 — Table S1. Baseline sociodemographic, socioeconomic, and lifestyle characteristics for the analytical and excluded samples. Table S2. Baseline sociodemographic, socioeconomic, and lifestyle characteristics for the analytical sample and those participants initially interviewed in the baseline phase of the Seniors‐ENRICA‐2 cohort who did not participate in the follow‐up phases. [file JOIM-298-123-s001.docx]

**Appendix**

For the multilevel mixed effects logistics models to assess the prospective association between plasma concentrations of amino acids and healthy aging, the following statistical model was used for the fully-adjusted model in each case.

$$logit \left( \pi_{j} \right)=\beta_{0}+\beta_{1}*{AminoAcid}_{j}+\beta_{2}*{Age}_{j}+\beta_{3}*{Sex}_{j}+\beta_{4}*{Education}_{j}+\beta_{5}*{Occupation}_{j}+\beta_{6}*{PhysicalActivity}_{j}+\beta_{7}*{BMI}_{j}+\beta_{8}*{TobaccoConsumption}_{j}+\beta_{9}*{SleepDuration}_{j}+\mu_{j}$$

where:

$\pi_{j}$ = Probability of healthy aging at time *j*.

*j* = Baseline, 2019 (1^st^ follow-up), 2022 (2^nd^ follow-up).

*µ_j_* = Cluster-level random effect at time *j*.

| **Supplementary Table S1.** Baseline sociodemographic, socioeconomic, and lifestyle characteristics for the analytical and excluded samples. | | | | | | | | | | | | | | | | | | | | | | |
| --- | --- | --- | --- | --- | --- | --- | --- | --- | --- | --- | --- | --- | --- | --- | --- | --- | --- | --- | --- | --- | --- | --- |
| **Variables** | | **Analytical sample**  (n = 859) | | | | | | | | | | **Excluded sample^*^**  (n = 368) | | | | | | | | | *p-value***^1^** | |
|  |  |  |  |  |  |  |  |  | |  |  | |  |  |  |  |  |  | | | |  |
| **Age (years), Mean (SD)** | | | | | | 70.9 (4.0) | | |  | | | | | 71.6 (4.3) | | | | |  | 0.008 | | |
| **Sex, n (%)** | | | | | |  | | |  | | | | |  | | | | |  | 0.169 | | |
| Men | | | | | | 443 (51.6) | | |  | | | | | 174 (47.3) | | | | |  |  | | |
| Women | | | | | | 416 (48.4) | | |  | | | | | 194 (52.7) | | | | |  |  | | |
| **Educational attainment, n (%)** | | | | | |  | | |  | | | | |  | | | | |  | 0.826 | | |
| University | | | | | | 252 (29.3) | | |  | | | | | 106 (28.8) | | | | |  |  | | |
| Secondary school | | | | | | 407 (47.4) | | |  | | | | | 181 (49.2) | | | | |  |  | | |
| Primary education or less | | | | | | 200 (23.3) | | |  | | | | | 81 (22.0) | | | | |  |  | | |
| **Householder's occupational**  **category, n (%)** | | | | | |  | | |  | | | | |  | | | | |  | 0.051 | | |
| Professionals and managers | | | | | | 250 (29.1) | | |  | | | | | 79 (21.5) | | | | |  |  | | |
| Lower non-manual workers | | | | | | 316 (36.8) | | |  | | | | | 154 (41.9) | | | | |  |  | | |
| Skilled manual workers | | | | | | 203 (23.6) | | |  | | | | | 90 (24.5) | | | | |  |  | | |
| Unskilled manual workers | | | | | | 90 (10.5) | | |  | | | | | 43 (11.7) | | | | |  |  | | |
| **Physical activity (METs h/week),**  **Mean (SD)** | | | | | | 69.0 (36.6) | | |  | | | | | 68.7 (38.0) | | | | |  | 0.876 | | |
| **BMI (kg/m^2^), Mean (SD)** | | | | | | 27.3 (4.2) | | |  | | | | | 28.1 (4.7) | | | | |  | 0.004 | | |
| **Smoking status, n (%)** | | | | | |  | | |  | | | | |  | | | | |  | 0.722 | | |
| Current smokers | | | | | | 425 (49.5) | | |  | | | | | 178 (48.4) | | | | |  |  | | |
| Never smokers | | | | | | 434 (50.5) | | |  | | | | | 190 (51.6) | | | | |  |  | | |
| **Sleep duration, n (%)** | | | | | |  | | |  | | | | |  | | | | |  | 0.885 | | |
| Non-optimal | | | | | | 515 (60.0) | | |  | | | | | 219 (59.5) | | | | |  |  | | |
| Optimal | | | | | | 344 (40.1) | | |  | | | | | 149 (40.5) | | | | |  |  | | |
| **AHEI-2010 score, Mean (SD)** | | | | | | 63.7 (9.7) | | |  | | | | | 62.6 (9.9) | | | | |  | 0.066 | | |

^*^Participants in the five-year follow-up period without plasma samples or observed values in the remaining study variables.

*^1^p*-values were obtained after applying an unpaired t test or chi-square test, depending on the type of variable.

**Notes**: SD: *Standard deviation;* MET*: Metabolic Equivalent Task;* BMI*: Body Mass Index;* AHEI*: Alternate Healthy Eating Index*.

| **Supplementary Table S2.** Baseline sociodemographic, socioeconomic, and lifestyle characteristics for the analytical sample and those participants initially interviewed in the baseline phase of the Seniors-ENRICA-2 cohort who did not participate in the follow-up phases. | | | | | | | | | | | | | | | | | | | | | | | | | |
| --- | --- | --- | --- | --- | --- | --- | --- | --- | --- | --- | --- | --- | --- | --- | --- | --- | --- | --- | --- | --- | --- | --- | --- | --- | --- |
| **Variables** | | | **Analytical sample**  (n = 859) | | | | | | | | | | | | **Excluded sample^*^**  (n = 2,414) | | | | | | | | *p-value***^1^** | | |
|  |  | |  |  |  |  | |  |  |  | | |  |  | |  |  |  |  |  |  | | | |  |
| **Age (years), Mean (SD)** | | | | | | | 70.9 (4.0) | | | |  | | | | 72.2 (4.6) | | | | | | |  | | <0.001 | |
| **Sex, n (%)** | | | | | | |  | | | |  | | | |  | | | | | | |  | | 0.001 | |
| Men | | | | | | | 443 (51.6) | | | |  | | | | 1,091 (45.2) | | | | | | |  | |  | |
| Women | | | | | | | 416 (48.4) | | | |  | | | | 1,323 (54.8) | | | | | | |  | |  | |
| **Educational attainment, n (%)** | | | | | | |  | | | |  | | | |  | | | | | | |  | | 0.012 | |
| University | | | | | | | 252 (29.3) | | | |  | | | | 575 (23.8) | | | | | | |  | |  | |
| Secondary school | | | | | | | 407 (47.4) | | | |  | | | | 1,227 (50.8) | | | | | | |  | |  | |
| Primary education or less | | | | | | | 200 (23.3) | | | |  | | | | 610 (25.3) | | | | | | |  | |  | |
| **Householder's occupational**  **category, n (%)** | | | | | | |  | | | |  | | | |  | | | | | | |  | | <0.001 | |
| Professionals and managers | | | | | | | 250 (29.1) | | | |  | | | | 486 (20.1) | | | | | | |  | |  | |
| Lower non-manual workers | | | | | | | 316 (36.8) | | | |  | | | | 876 (36.3) | | | | | | |  | |  | |
| Skilled manual workers | | | | | | | 203 (23.6) | | | |  | | | | 654 (27.1) | | | | | | |  | |  | |
| Unskilled manual workers | | | | | | | 90 (10.5) | | | |  | | | | 376 (15.6) | | | | | | |  | |  | |
| **Physical activity (METs h/week),**  **Mean (SD)** | | | | | | | 69.0 (36.6) | | | |  | | | | 65.6 (36.1) | | | | | | |  | | 0.017 | |
| **BMI (kg/m^2^), Mean (SD)** | | | | | | | 27.3 (4.2) | | | |  | | | | 27.3 (4.2) | | | | | | |  | | <0.001 | |
| **Smoking status, n (%)** | | | | | | |  | | | |  | | | |  | | | | | | |  | | 0.097 | |
| Current smokers | | | | | | | 425 (49.5) | | | |  | | | | 1,115 (46.2) | | | | | | |  | |  | |
| Never smokers | | | | | | | 434 (50.5) | | | |  | | | | 1,299 (53.8) | | | | | | |  | |  | |
| **Sleep duration, n (%)** | | | | | | |  | | | |  | | | |  | | | | | | |  | | 0.178 | |
| Non-optimal | | | | | | | 515 (60.0) | | | |  | | | | 1,510 (62.6) | | | | | | |  | |  | |
| Optimal | | | | | | | 344 (40.1) | | | |  | | | | 904 (37.5) | | | | | | |  | |  | |
| **AHEI-2010 score, Mean (SD)** | | | | | | | 63.7 (9.7) | | | |  | | | | 62.1 (9.3) | | | | | | |  | | <0.001 | |
|  | |  | | | | | | | | |  |  | | | | | | | | | |  | |  | |

^*^Those who were interviewed in the baseline phase of the Seniors-ENRICA-2 cohort, but did not participate in the follow-up phases.

*^1^p*-values were obtained after applying an unpaired t test or chi-square test, depending on the type of variable.

**Notes**: SD: *Standard deviation;* MET*: Metabolic Equivalent Task;* BMI*: Body Mass Index;* AHEI*: Alternate Healthy Eating Index*.
